# Aniridia‐associated keratopathy: Clinical and molecular mechanisms of disease progression and emerging therapeutic targets

**DOI:** 10.1111/aos.70086

**Published:** 2026-02-03

**Authors:** N. Szentmáry, S. Suiwal, M. Amini, F. N. Fries, A. Náray, L. Latta, Z. Li, S. Li, S. Liu, S. L. Hsu, S. Kundu, G. Tóth, S. Trusen, J. Zimmermann, B. Seitz, B. Käsmann‐Kellner, Z. Hoxha, A. Langenbucher, O. Stachs, M. Cortón‐Perez, K. Tory, A. Csorba, Z. Z. Nagy, M. C. Acosta, J. Gallar, M. Csidey, E. Maka, T. Stachon

**Affiliations:** ^1^ Dr. Rolf M. Schwiete Center for Limbal Stem Cell and Congenital Aniridia Research, Saarland University Homburg/Saar Germany; ^2^ Department of Ophthalmology Semmelweis University Budapest Hungary; ^3^ Experimental Ophthalmology Saarland University Homburg/Saar Germany; ^4^ Department of Ophthalmology Saarland University Medical Center Homburg/Saar Germany; ^5^ Department of Ophthalmology Rostock University Medical Center Rostock Germany; ^6^ Department Life, Light & Matter University Rostock Rostock Germany; ^7^ Department of Genetics and Genomics Instituto de Investigación Sanitaria‐Fundación Jiménez Díaz University Hospital, Universidad Autónoma de Madrid (IIS‐FJD, UAM) Madrid Spain; ^8^ Center for Biomedical Network Research on Rare Diseases (CIBERER), Instituto de Salud Carlos III Madrid Spain; ^9^ 1st Department of Pediatrics Semmelweis University Budapest Hungary; ^10^ Instituto de Neurociencias Universidad Miguel Hernández‐CSIC San Juan de Alicante Spain; ^11^ Instituto de Investigación Sanitaria y Biomédica de Alicante (ISABIAL) Alicante Spain; ^12^ Department of Opthalmology Budapest Heim Pál National Pediatric Institute Budapest Hungary

## Abstract

Congenital aniridia is a rare genetic disorder primarily caused by pathogenic variants of the PAX6 gene. It leads to various panocular anomalies, including aniridia‐associated keratopathy (AAK). This review highlights recent insights into its pathogenesis, focusing on clinical staging, microstructural changes in the cornea and molecular dysregulation. We synthesized clinical and experimental findings from large European cohorts, integrating data on over 550 eyes. AAK severity correlates with iris malformation, secondary glaucoma and lens status. In vivo confocal microscopy reveals a reduction in subbasal nerve plexus density, altered keratocyte and endothelial morphology and an increase in Langerhans cell infiltration. RNA and miRNA microarrays, as well as RNA‐seq studies, highlight dysregulated miRNAs (such as miR‐204‐5p and miR‐138‐5p) and altered expression of PAX6 and keratocyte markers. Limbal fibroblasts show enhanced inflammatory responses and vulnerability to oxidative stress. Advanced AAK is associated with a reduced quality of life. The progression of AAK involves intricate interactions between developmental deficits, inflammation and changes in the limbal microenvironment, suggesting molecular targets for future therapies.

## 1 INTRODUCTION

Congenital aniridia is a rare, bilateral, panocular disorder characterized by partial or near‐total absence of the iris, affecting approximately 1 in 40 000 to 1 in 100 000 individuals (Hall et al., 2025; Latta, Figueiredo, et al., 2021; Latta, Ludwig, et al., 2021). The disease is most frequently inherited in an autosomal dominant pattern due to haploinsufficiency of the *PAX6* gene, a master regulator of ocular development. More than 700 pathogenic variants in *PAX6*, spanning both coding and non‐coding regions, have been documented, underscoring the genetic complexity of this condition (Hall et al., 2025). While the iris anomalies are the most visually striking feature, aniridia is a spectrum disease that compromises the structure and function of nearly all ocular tissues (Hall et al., 2025; Latta, Figueiredo, et al., 2021; Latta, Ludwig, et al., 2021). Patients often present with aniridia‐associated keratopathy (AAK), cataract, secondary glaucoma, foveal hypoplasia, optic nerve head anomalies and nystagmus, all of which contribute to reduced visual acuity from early life (Fries, Náray, et al., 2024; Fries et al., 2023. In addition, *PAX6* haploinsufficiency may also have effects on metabolic balance, cardiovascular health and the central nervous system (Obst et al., 2025).

Among ocular complications, AAK is a progressive corneal disease characterized by limbal stem cell deficiency (LSCD), recurrent epithelial breakdown, corneal vascularization and stromal scarring, ultimately threatening vision and ocular integrity. Studies report that up to 80% of individuals with aniridia develop some degree of AAK during their lifetime (Fries, Náray, et al., 2024; Fries, Obst, et al., 2024; Náray et al., 2023).

The pathogenesis of AAK is multifactorial, involving both intrinsic developmental defects (Latta, Figueiredo, et al., 2021; Latta, Ludwig, et al., 2021) and extrinsic factors such as inflammation (Amini et al., 2025; Kundu et al., 2025) and mechanical stress with ocular surface irregularities (Náray et al., 2023). *PAX6* pathogenic variants disrupt limbal epithelial stem cell maintenance and corneal epithelial differentiation, predisposing the ocular surface to chronic instability (Latta, Figueiredo, et al., 2021; Latta, Ludwig, et al., 2021; Li et al., 2025a, 2025b, 2025c, 2025d). In addition, recent investigations have highlighted the role of corneal nerve alterations (Csorba et al., 2024; Lagali et al., 2020), stromal fibroblast changes (Hsu et al., 2025; Li et al., 2025a, 2025b, 2025c, 2025d; Liu et al., 2025; Trusen et al., 2024), specific endothelial layer characteristics (Csidey et al., 2025) and an altered immune (Moustardas et al., 2024) and inflammatory (Amini et al., 2025; Kundu et al., 2025) microenvironment in driving disease progression.

Emerging molecular studies provide further insight into AAK pathophysiology. Dysregulation of key microRNAs (Latta, Figueiredo, et al., 2021; Latta, Ludwig, et al., 2021) such as miR‐204‐5p (Abbasi et al., 2024; Amini et al., 2025; Latta, Figueiredo, et al., 2021; Latta, Ludwig, et al., 2021) and miR‐138‐5p (Nastaranpour et al., 2025) has been implicated in aberrant angiogenesis and inflammatory pathways. Moreover, transcriptomic profiling of limbal epithelial and stromal cells from aniridia patients reveals disrupted signalling networks, including pathways mediated by interleukins and TNF, which may exacerbate ocular surface and corneal disease (Stachon, unpublished).

Given the complexity of AAK and its significant impact on visual prognosis and quality of life, there is a pressing need to integrate clinical, imaging and molecular data to develop more effective management strategies. This review aims to provide a comprehensive overview of the clinical manifestations, confocal microscopic and molecular changes associated with AAK and to discuss emerging therapeutic approaches that target the underlying disease mechanisms.

## 2 AAK STAGES AND THEIR CLINICAL ASSOCIATIONS

**FIGURE 1:**
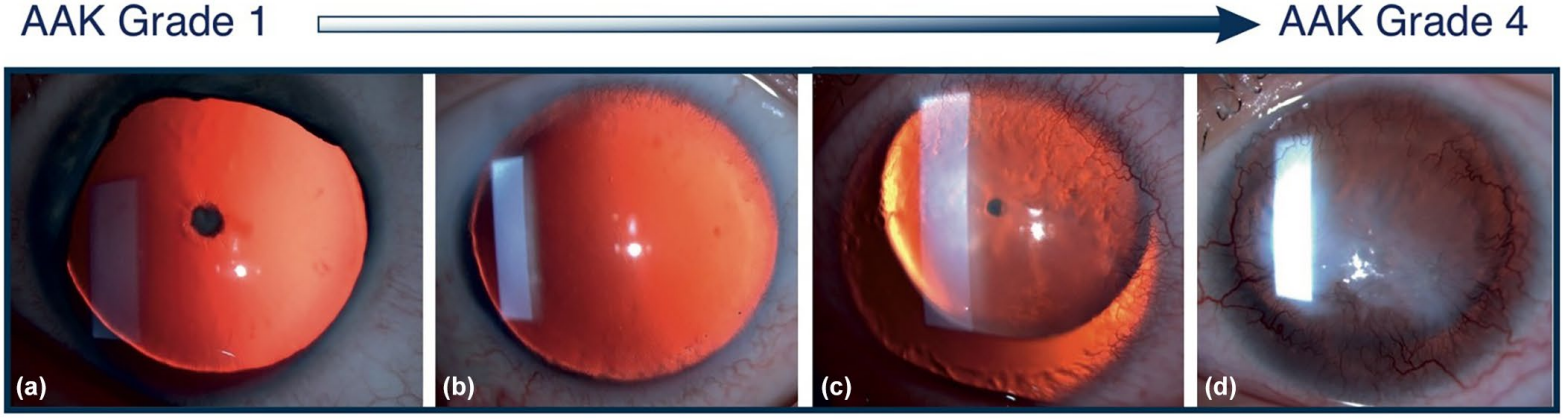
Clinical and surgical factors associated with or triggering the progression of aniridia‐associated keratopathy (AAK) from Lagali Grade 1 (A) to Grades 2–4 (B‐D) (Lagali et al., 2018). Factors contributing to AAK advancement include: (1) specific pathogenic variants of the PAX6 gene; (2) the severity of iris malformation; (3) ocular surface inflammation induced by external stimuli; (4) an increased number of pressure‐lowering medications required for glaucoma management; (5) lens status, with more advanced AAK observed in aphakic and pseudophakic eyes; (6) a history of glaucoma surgery, such as trabeculectomy or tube shunt implantation; (7) previous cataract surgery, identified as a significant trigger of AAK progression; and (8) Morcher intraocular lens or artificial iris implantation, which are associated with more severe AAK compared to in‐the‐bag lens implantation (Fries, Náray, et al., 2024; Fries, Obst, et al., 2024; Hall et al., 2025; Náray et al., 2025; Tóth et al., 2025).

AAK follows a characteristic clinical course, usually beginning with subtle limbal alterations and ocular surface inflammation, and progressively advancing to more severe stages of LSCD (Latta, Figueiredo, et al., 2021; Latta, Ludwig, et al., 2021). This is accompanied by conjunctivalization advancing from the corneal periphery towards the centre, neovascularization, recurrent epithelial breakdown and ultimately stromal scarring and pannus formation, all of which compromise corneal transparency. According to Mackman's classification, stages 0, 1A, 1B and 2 were identified; according to Mayer, stages I‐V; in López‐García's system, stages 1 to 3 (Náray et al., 2023). Recently, Lagali et al. introduced a structured staging system ranging from grade 0 (no detectable keratopathy) to grade 4 (severe, total LSCD with opaque vascularized cornea), which has become the benchmark for assessing disease severity and guiding management decisions (Lagali et al., 2020) (Figure 1). In a previous paper, Lagali and colleagues also proposed an additional Grade 5 AAK, representing an even more advanced stage of the disease, and several subsequent studies have adopted this extended classification (Lagali et al., 2018).

Extensive data from the Homburg Aniridia Center encompassing 556 eyes provide valuable epidemiologic insight into the natural history of AAK. This cross‐sectional analysis, performed in a cohort with a mean age of 20.1 ± 20.1 years, revealed that Stage 1 disease—characterized by peripheral vascular ingrowth extending less than 1 mm beyond the limbus and mild epithelial irregularities—was the most common presentation (32%). Stage 2 disease followed in prevalence (19%), showing more extensive limbal involvement while sparing the central 2–3 mm of the cornea and displaying superficial neovascularization. Advanced stages—Stage 3 (12%), Stage 4 (11%) and Stage 5 (8%)—together represented more than 30% of affected eyes, showing the progressive and chronic nature of AAK in a substantial subset of patients. Importantly, the severity of iris malformations exhibited a robust correlation with higher AAK grades, as well as with the occurrence of secondary glaucoma and lens opacities (*p* < 0.001 for all associations), highlighting how developmental anterior segment anomalies often cluster together and compound disease burden (Fries, Náray, et al., 2024; Fries, Obst, et al., 2024).

Secondary glaucoma was documented in approximately 55% of cases at the Homburg Aniridia Center, necessitating aggressive management strategies such as multi‐agent topical therapy or surgical intervention (Fries et al., 2023). Both increased numbers of pressure‐lowering medications and histories of trabeculectomy or tube shunt implantation were statistically associated with higher AAK stages, suggesting either a common pathogenic pathway involving PAX6‐driven structural fragility or additive iatrogenic contributions from chronic medication toxicity and surgical alteration of the ocular milieu (Fries et al., 2023; Liu et al., 2025). This linkage of anterior segment dysgenesis, intraocular pressure elevation and corneal surface deterioration illustrates the complex clinical landscape faced by patients and clinicians alike.

In 553 eyes of 286 individuals with congenital aniridia, lens status showed a clear impact on visual acuity and intraocular pressure. It was closely linked to the severity of AAK and the presence of glaucoma. Across different age groups, advanced AAK was most consistently observed in pseudophakic and aphakic eyes, while glaucoma was particularly associated with pseudophakia, aphakia and lens subluxation. These findings suggest that cataract surgery in congenital aniridia is strongly associated with progression of AAK and secondary glaucoma, highlighting the need for particular caution when considering surgical intervention (Náray et al., 2025). In addition, eyes that had undergone Morcher intraocular lens implantation or artificial iris implantation exhibited more advanced AAK stages and a higher incidence of glaucoma compared with eyes in which the IOL was implanted within the capsular bag. Therefore, Morcher IOL and artificial iris implantations should be avoided in patients with congenital aniridia (Náray et al., 2025).

## 3 CORNEAL MICROSTRUCTURAL ALTERATIONS

### 3.1 Corneal sensitivity and sub‐basal nerve plexus changes

Recent investigation described that patients with congenital aniridia exhibit reduced corneal sensitivity, particularly to mechanical and cold stimuli, most notably in adults and those with advanced AAK or loss‐of‐function mutations. Reflex tearing is often diminished with a lower tearing reserve, while basal tearing and blinking remain similar to controls. These findings suggest progressive dysfunction of corneal sensory receptors, worsening with age and disease severity (Acosta, unpublished).

In vivo confocal microscopy (IVCM) has emerged as an indispensable modality for elucidating the microstructural corneal changes, also associated with congenital aniridia. Through high‐resolution, non‐invasive imaging of the corneal subbasal nerve plexus (SBNP), IVCM provides critical insights that would be unattainable with traditional slit‐lamp biomicroscopy alone. Studies using this technique report significant reductions in SBNP density in patients with congenital aniridia, supporting the concept of a congenital corneal neuropathy arising from disrupted ocular development due to *PAX6* haploinsufficiency, further exacerbated by chronic, inflammation‐driven neural alterations (Csorba et al., 2024; Lagali et al., 2020).

These reductions in corneal nerve fibre density have profound implications for the maintenance of a stable and healthy ocular surface. Corneal nerves play a pivotal role in providing essential trophic support to the epithelium, influencing cell proliferation, differentiation and wound healing through the release of neuropeptides such as substance P (Lasagni Vitar, Rama, & Ferrari, 2022; Lasagni Vitar, Triani, et al., 2022; Vitar et al., 2021) and calcitonin gene‐related peptide (CGRP) (Khan et al., 2002). A compromised nerve architecture thus predisposes the epithelium to recurrent erosions and delayed healing responses, phenomena frequently observed in AAK (Latta, Figueiredo, et al., 2021; Latta, Ludwig, et al., 2021).

Moreover, the loss of normal sensory innervation disrupts the intricate reflex arcs responsible for tear secretion, contributing to the high prevalence of dry eye symptoms and ocular surface discomfort reported by patients (Acosta, unpublished). Even at relatively early stages of AAK, many individuals describe fluctuating vision, photophobia and foreign body sensation, underscoring how neurotrophic deficits can manifest clinically before overt structural changes become apparent (Csorba et al., 2024; Lagali et al., 2020).

Interestingly, the pattern of corneal nerve loss observed in aniridia often appears diffuse rather than localized, consistent with a congenital aetiology rather than an acquired post‐inflammatory degeneration. This contrasts with secondary neurotrophic keratopathies seen in herpetic disease or diabetes, where regional loss is typical (Dana et al., 2021). Such observations further reinforce the notion that corneal neuropathy in aniridia is fundamentally developmental, intertwined with broader anomalies of the ocular surface microenvironment (Latta, Figueiredo, et al., 2021; Latta, Ludwig, et al., 2021). The corneal microenvironment—particularly chronic inflammation—may exacerbate pre‐existing neuropathy by triggering mild neuritis that further progresses to additional neural damage, thereby worsening nerve dysfunction in congenital aniridia (Csorba et al., 2024; Fries et al., 2022).

In addition to quantitative reductions, qualitative changes in nerve morphology have also been noted, including increased tortuosity and abnormal branching patterns (Csorba et al., 2024). These alterations may reflect compensatory attempts at regeneration or incomplete maturation processes during corneal development. Taken together, these findings highlight the crucial role of intact corneal innervation in preserving epithelial integrity and underscore the potential of neuroprotective and neuroregenerative strategies to mitigate the progression of AAK (Csorba et al., 2024). Early suppression of inflammation at a potential subclinical stage of keratopathy represents another promising therapeutic target that warrants further investigation (Fries et al., 2022).

### 3.2 Stromal and Langerhans cell pathology

Stromal abnormalities in congenital aniridia are equally pronounced and represent a critical component of the complex pathophysiology underlying AAK. IVCM studies have demonstrated a significant reduction in keratocyte densities within the central and anterior stroma. This decrease is accompanied by distinct morphological alterations, including elongated cellular processes and the formation of confluent clusters of anterior keratocytes that appear disorganized compared to the orderly arrangement observed in healthy corneas (Csorba et al., 2024; Lagali et al., 2020).

These stromal changes may have several functional consequences. Keratocytes are essential not only for maintaining stromal transparency through regulated synthesis and remodelling of the extracellular matrix but also play an active role in wound healing responses. Alterations in keratocyte density and morphology could thus contribute to the increased propensity for stromal haze and scarring observed in advanced stages of AAK, particularly following minor epithelial insults that might otherwise heal without sequelae in a normal cornea (Csorba et al., 2024).

Another striking feature identified in IVCM studies of aniridia is the presence of hyperreflective punctate deposits scattered throughout the stromal layers. While the precise biochemical composition of these microdots remains to be fully elucidated, it has been hypothesized that they may represent accumulations of metabolic byproducts or extracellular matrix fragments arising from disrupted stromal homeostasis. Alternatively, these deposits might reflect localized regions of stromal stress or early degenerative changes linked to chronic subclinical inflammation (Csorba et al., 2024).

Adding to the complexity of the stromal microenvironment in aniridia is the observed increase in both the density and maturity of Langerhans cells (LCs) within the cornea. Under physiological conditions, LCs are sparsely distributed and typically reside in a relatively immature state within the peripheral cornea, contributing to its immune‐privileged status. However, with advancing AAK stage, there is a notable centripetal migration of LCs coupled with morphological maturation from Stage 2 to Stage 3 keratopathy, as evidenced by the development of longer dendritic processes. This pattern strongly suggests an evolving pro‐inflammatory milieu within the corneal stroma (Csorba et al., 2024). Such immune cell activation likely plays a dual detrimental role: directly promoting chronic low‐grade inflammation that destabilizes the epithelial barrier and facilitating the ingrowth of conjunctival vessels and fibrovascular pannus, hallmark features of progressive AAK. The interaction between altered keratocyte function, metabolic disturbances signalled by stromal deposits and heightened LC activity underscores a vicious cycle wherein structural abnormalities and immune dysregulation perpetuate each other, driving disease progression even in the absence of overt infection or injury. This highlights the potential importance of anti‐inflammatory and immunomodulatory strategies in the management of early to moderate AAK to disrupt this deleterious cascade.

### 3.3 Endothelial findings

The corneal endothelial layer in congenital aniridia presents a more complex and somewhat paradoxical picture compared to the more straightforward disruptions seen in the epithelium and stroma. Some observational studies have reported that corneal endothelial cells in patients with aniridia, particularly in earlier disease stages or younger individuals, appear to maintain a relatively preserved morphology and may even exhibit slightly higher cell densities than those found in age‐matched healthy controls (Csidey et al., 2025; Lagali et al., 2020). In the analysis by Csidey et al., most endothelial parameters, including cell density, cell area, polygonality and hexagonality, did not differ between aniridia and healthy eyes. However, mean endothelial cell diameter and the Clark‐Evans‐Index (CE‐Index) were significantly lower in aniridia, suggesting subtle qualitative differences with slightly better endothelial quality and greater reserves. Age‐related correlations of endothelial parameters in aniridia mirrored those reported in healthy eyes, reflecting normal age‐dependent remodelling in a nonregenerative tissue (Csidey et al., 2025).

A striking finding is the presence of endothelial deposits exclusively in aniridia eyes, correlating with age, AAK grade, CE‐Index and stromal microdots, but not with iris malformation, cataract, glaucoma, or keratocyte density (Csidey et al., 2025). Their characteristics suggest either a metabolic alteration specific to *PAX6*‐related aniridia or a manifestation of ocular surface inflammation, in line with reports of increased inflammatory cytokines and corneal inflammatory cells in advanced AAK (Landsend et al., 2018; Stachon et al., 2024).

Interestingly, eyes with congenital anterior polar cataract display superior endothelial parameters, reinforcing a developmental link between lens and corneal endothelium, likely rooted in surface ectoderm and neural crest interactions influenced by *PAX6*. In glaucoma, alpha‐adrenergic therapy has a significant effect on endothelial measures, similar to other glaucomatous eyes. In addition, intraocular surgery, particularly cataract extraction, is associated with typical endothelial cell loss in congenital aniridia (Csidey et al., 2025).

Altogether, in congenital aniridia, the corneal endothelium may exhibit slightly higher quality and greater reserve capacity compared to healthy individuals, possibly reflecting developmental abnormalities associated with *PAX6* pathogenic variants. In contrast, endothelial deposits appear unrelated to developmental defects and may instead be linked to yet undescribed inflammatory or metabolic alterations of the endothelium in *PAX6*‐related congenital aniridia (Csidey et al., 2025). Recognizing these endothelial nuances emphasizes the need for careful perioperative planning and long‐term surveillance in managing aniridic eyes, underscoring that even layers appearing relatively spared on cursory examination may harbour latent vulnerabilities integral to the disease process.

## 4 MOLECULAR AND CELLULAR PATHOPHYSIOLOGY

### 4.1 Limbal epithelial stem cells and PAX6

At the molecular level, *PAX6* stands as the quintessential master transcription factor governing ocular development, exerting its influence across every tissue of the eye. From orchestrating early morphogenesis of the optic cup to directing the maturation of specialized cell types, *PAX6*'s regulatory reach is profound. Its role in maintaining the limbal epithelial stem cell (LESC) niche is particularly critical, as this population of cells at the corneoscleral junction is indispensable for lifelong renewal of the corneal epithelium (Latta, Figueiredo, et al., 2021; Latta, Ludwig, et al., 2021).

In congenital aniridia, *PAX6* haploinsufficiency disrupts this finely tuned system, compromising both the establishment and ongoing maintenance of the LESC population. This molecular deficiency disturbs the delicate homeostatic balance required for orderly corneal epithelial turnover and stratification. Studies using patient‐derived limbal epithelial cells (LECs) have demonstrated markedly reduced expression of structural and differentiation markers, most notably keratins K3 and K12, which are hallmarks of the mature corneal epithelial phenotype (Latta et al., 2018; Schlötzer‐Schrehardt et al., 2021). The reduction in these keratins underscores the shift towards a more conjunctivalized epithelial state.

Beyond keratins, PAX6 insufficiency also impacts metabolic pathways essential for epithelial health. Levels of enzymes such as alcohol dehydrogenase 7 (ADH7) and aldehyde dehydrogenase 1 family member A1 (ALDH1A1), key players in the retinoic acid signalling axis, are significantly diminished (Latta et al., 2019). This pathway is vital for epithelial differentiation and wound healing, suggesting that PAX6 deficiency triggers a cascade of transcriptional disturbances extending well beyond structural proteins. Disruption of retinoic acid metabolism could help explain the predisposition for persistent epithelial defects and delayed re‐epithelialization observed in AAK.

Studies using siRNA‐mediated PAX6 knockdown in primary limbal cell cultures have validated these findings. This experimental model successfully recapitulated the molecular phenotype observed in patient tissues, faithfully reproducing the downregulation of keratin 3 and 12 (KRT3 and KRT12), ADH7 and ALDH1A1, thereby highlighting the direct regulatory influence of PAX6 on these genes (Latta et al., 2019). There are several further epithelial cell models with the reduced PAX6 level. A PAX6^+/−^ aniridia cell line, established through the introduction of an AAK‐associated nonsense mutation using CRISPR/Cas9, provides a reproducible platform to study the direct molecular effects of this mutation under standardized conditions (Roux et al., 2018). Additionally, induced pluripotent stem cells (iPSCs) generated from aniridia patients offer another valuable system for exploring disease mechanisms (Ilmarinen et al., 2023). Furthermore, patient‐derived immortalized LECs provide a valuable in vitro model for studying the molecular and cellular mechanisms underlying congenital aniridia (Stachon, unpublished). Such experimental systems serve as powerful tools not only for elucidating disease mechanisms but also for identifying and testing therapeutic compounds capable of restoring or compensating for disrupted transcriptional networks (Li et al., 2025a, 2025b, 2025c, 2025d).

Interestingly, fatty acid binding protein 5 (FABP5), a downstream target of PAX6, plays a previously unrecognized role in the progression of AAK by modulating the expression of genes and proteins critical for LEC differentiation. Moreover, under inflammatory conditions, FABP5 knockdown alters the activity of key transcription factors (*PAX6*, fos‐related antigen 2 (*FOSL2*)), as well as genes associated with LEC migration, differentiation and cellular homeostasis [*KRT3*, vascular endothelial growth factor A (*VEGFA*), MAP kinase‐activated protein kinase 3 (*MAPK3*), cellular retinoic acid binding protein 2 (*CRABP2*)], and those mediating inflammatory responses [interleukin‐6 (*IL‐6*), prostaglandin E synthase 2 (*PTGES2*)]. Given its central role in coordinating epithelial differentiation and inflammatory signalling, FABP5 represents a promising molecular target for therapeutic intervention in congenital aniridia (Amini, unpublished).

Furthermore, PAX6 insufficiency disrupts apoptotic signalling in LECs by suppressing pro‐apoptotic mediators and enhancing cell survival pathways. These findings underscore the pivotal role of PAX6 in regulating LEC apoptosis and cellular stress adaptation, while also identifying potential molecular targets for future therapeutic investigation (Liu, unpublished).

From a developmental perspective, this fundamental compromise in *PAX6*‐driven gene expression sets the stage for a limbal niche that is both quantitatively depleted in stem cells and qualitatively deficient in its supportive microenvironment. The net result is a surface inherently prone to instability, unable to mount robust regenerative responses and vulnerable to encroachment by conjunctival epithelium and neovessels—a pathophysiological sequence that defines the progression of LSCD and AAK (Latta et al., 2019).

Looking ahead, these insights highlight PAX6 as a potential molecular target for future therapies. Approaches that could upregulate residual PAX6 activity, modulate its downstream effectors, or stabilize the expression of critical corneal differentiation markers may hold promise in mitigating the progression of AAK (Avila et al., 2025; Dorot et al., 2021; Moustardas et al., 2024; Oved et al., 2021; Suiwal et al., 2025). Furthermore, modulating FABP5 activity could help restore epithelial homeostasis, stabilize limbal stem cell function and mitigate inflammation‐driven progression of AAK (Amini, unpublished). Targeting the dysregulated apoptotic pathways may also represent a promising therapeutic strategy to preserve LECs' viability and slow the progression of AAK (Liu, unpublished). Thus, understanding the multifaceted role of PAX6 in governing the limbal niche not only illuminates the pathogenesis of ocular surface disease in congenital aniridia but also opens avenues for innovative molecular interventions.

### 4.2 Stromal cell dysfunction

The limbal stroma is increasingly recognized as a dynamic participant in ocular surface health, far more than a mere passive scaffold supporting overlying epithelial layers. This mesenchymal compartment plays critical roles in regulating the limbal stem cell niche, maintaining extracellular matrix integrity and modulating local immune responses. In the context of congenital aniridia, recent investigations have shed light on profound alterations in the functional phenotype of limbal stromal cells, with important implications for the pathogenesis and chronicity of AAK (Li et al., 2025a, 2025b, 2025c, 2025d). *PAX6* mRNA level is reduced in aniridia‐derived stromal fibroblasts compared to controls. Furthermore, aniridia fibroblasts show lower collagen type V alpha 1 chain (*COL5A1*) levels and higher aldehyde dehydrogenase 3A1 (*ALDH3A1*) and *keratin* expression, while in another, keratocyte‐phenotype triggering culture medium both collagen type I and type V alpha 1 chain (*COL1A1* and *COL5A1*) are increased, accompanied by elevated COL1A1 and alpha‐smooth muscle actin (α‐SMA) protein levels. These findings highlight altered PAX6 and keratocyte‐specific marker regulation in aniridia stromal cells, suggesting a role in the development of AAK (Li et al., 2025a, 2025b, 2025c, 2025d).

Studies using primary cultures of limbal fibroblasts derived from aniridic patients have demonstrated that these cells exhibit exaggerated responses to inflammatory stimuli. When challenged with bacterial lipopolysaccharide (LPS), a prototypical activator of innate immune pathways, aniridia fibroblasts show markedly elevated expression of pro‐inflammatory cytokines, particularly interleukin‐1β (IL‐1β) and interleukin‐6 (IL‐6). These mediators are known to amplify local inflammation, promote leukocyte infiltration and destabilize epithelial junctions, thereby facilitating recurrent epithelial defects and sustaining a hostile microenvironment that impedes effective wound healing (Zimmermann et al., 2025)

Moreover, the vulnerability of these stromal cells extends beyond exaggerated inflammatory reactivity. Under conditions of oxidative stress induced by cobalt chloride, an established hypoxia‐mimetic agent, aniridia‐derived fibroblasts display heightened activation of the nuclear factor erythroid 2–related factor 2 (Nrf2) pathway, a master regulator of cellular antioxidant defenses. This is accompanied by upregulated expression of downstream targets such as superoxide dismutase 1 (SOD1) and peroxiredoxin 6 (PRDX6), suggesting a compensatory attempt to counteract increased oxidative burden. While on the surface this might appear protective, the need for such amplified antioxidant responses underscores a baseline state of oxidative vulnerability that could predispose the stroma to long‐term degenerative changes and matrix remodelling (Trusen et al., 2024).

Intriguingly, when these fibroblasts are co‐cultured with LECs, bidirectional alterations in gene expression profiles are observed. This cross‐talk highlights how disrupted epithelial–stromal signalling in aniridia may create a vicious cycle: dysfunctional stromal fibroblasts produce abnormal cytokine or matrix signals that impair epithelial stem cell function, while stressed or dysregulated epithelium releases mediators that further skew stromal behaviour. Such maladaptive interactions likely contribute to the perpetuation of LSCD and recurrent epithelial breakdown that are hallmarks of AAK (Berger et al., 2025).

These findings collectively shift the paradigm of AAK pathophysiology from being purely an epithelial stem cell defect to a broader disorder involving a dysfunctional limbal microenvironment, where stromal fibroblasts emerge as active drivers of disease progression (Li et al., 2024). This opens up potential new therapeutic strategies aimed at reprogramming pathological fibroblasts to support regeneration, modulating stromal inflammation and oxidative stress, or even restoring healthy epithelial–stromal communication. Understanding and targeting these deeper stromal contributions could prove pivotal in halting or reversing the course of AAK, moving beyond surface‐level treatments towards truly disease‐modifying interventions.

### 4.3 miRNA and transcriptome findings

In recent years, there has been growing interest in the complex non‐coding RNA networks that regulate ocular surface biology, reshaping our understanding of corneal homeostasis and disease. Within this expanding field, microRNAs (miRNAs) have emerged as pivotal post‐transcriptional regulators, orchestrating diverse cellular processes through their capacity to fine‐tune gene expression networks. In the context of congenital aniridia, microarray profiling and next‐generation sequencing approaches have unveiled a striking dysregulation of miRNA expression within both conjunctival and limbal epithelial cells, offering new molecular insights into the pathogenesis of AAK.

Of particular pathophysiological significance is the robust downregulation of miR‐204‐5p, a miRNA recognized for its anti‐angiogenic properties mediated through direct suppression of VEGFA and its receptor VEGFR2 (Abbasi et al., 2024; Amini et al., 2025; Latta, Figueiredo, et al., 2021; Latta, Ludwig, et al., 2021; Nastaranpour et al., 2025). The loss of miR‐204‐5p expression effectively dismantles a critical molecular checkpoint that normally restrains angiogenic signalling, thereby lowering the barrier to neovascular encroachment into the typically avascular corneal stroma. This shift not only facilitates pannus formation, a hallmark of progressive AAK, but also perpetuates a microenvironment prone to chronic inflammation and scarring (Abbasi et al., 2024; Amini et al., 2025).

Conversely, miR‐138‐5p is markedly upregulated in aniridic limbal epithelial cells, further tipping the molecular balance towards disease progression. Bioinformatic analyses and experimental validation have identified several key targets of miR‐138‐5p that are central to cell cycle regulation (e.g., CCND1), apoptotic pathways (e.g., CASP3) and cytoskeletal organization (e.g., ROCK2). The overexpression of this miRNA may disrupt the delicate equilibrium between epithelial proliferation, controlled apoptosis and migration—processes that are integral to effective wound healing and maintenance of a stable, transparent corneal surface (Suiwal, unpublished).

In addition to these miRNA alterations, comprehensive transcriptomic analyses of mRNA expression profiles in patient‐derived LECs and limbal fibroblasts have revealed robust enrichment in pathways associated with innate and adaptive immune responses. Notably, there is upregulation of genes within the interleukin‐17 (IL‐17) and tumour necrosis factor (TNF) signalling cascades, classical mediators of pro‐inflammatory activity that drive leukocyte recruitment, cytokine amplification and tissue remodelling. Such signatures underscore that beyond primary developmental deficiencies imposed by PAX6 haploinsufficiency, there exists an active inflammatory component that likely sustains and aggravates corneal surface pathology over time (Stachon, unpublished).

Notably, these molecular findings not only deepen our mechanistic understanding of AAK but also point towards novel therapeutic avenues. The possibility of restoring miR‐204‐5p levels through miRNA mimics or antagonizing miR‐138‐5p with targeted inhibitors represents a promising strategy to rebalance angiogenic and apoptotic signalling on the ocular surface. Similarly, pharmacologic or biologic agents aimed at modulating IL‐17 and TNF pathways could potentially interrupt the chronic inflammatory cycles that fuel LSCD and epithelial breakdown. Together, these advances in miRNA and transcriptomic profiling move the field closer to precision medicine approaches for aniridia, whereby interventions might be tailored to correct specific molecular disturbances, offering hope for more effective, mechanism‐based therapies that go beyond symptomatic management.

## 5 INFLUENCE OF GENETIC VARIANTS ON THE CLINICAL PHENOTYPE

The most common genetic basis of congenital aniridia lies in pathogenic variants of the *PAX6* gene, although alterations in other genes—such as *FOXC1, PITX2, CYP1B1, FOXD3, PITX3, CPAMD8, ITPR1, TENM3, TRIM44, COL4A1, CRYAA* and *PXDN*—have also been linked to the condition. These manifest with a spectrum of ocular features including iris anomalies, keratopathy, cataracts, glaucoma, foveal hypoplasia, nystagmus and optic nerve changes. Accurate diagnosis requires distinguishing aniridia from conditions with overlapping features such as WAGR (Wilms tumour–Aniridia–Genitourinary anomalies–intellectual disability/Retardation), Axenfeld‐Rieger, ring‐chromosome 6, COL4A1‐related anterior segment dysgenesis, Gillespie syndrome, or Peters anomaly. Careful clinical evaluation combined with genetic testing is key to precise diagnosis and paves the way for tailored management strategies that can improve visual outcomes (Hall et al., 2025).

## 6 QUALITY OF LIFE IMPLICATIONS

While detailed structural and molecular studies elegantly chart the biological underpinnings of AAK, patient‐centred outcomes offer indispensable insight into the lived experience of this chronic disease, revealing how corneal pathology translates into tangible daily challenges. Standardized assessments of health‐related quality of life (HRQoL) conducted at Saarland University provide a nuanced picture: despite often markedly reduced best‐corrected visual acuity (BCVA) from an early age, many paediatric patients report satisfactory levels of overall life quality. This somewhat counterintuitive finding may be attributable to a combination of neuroplastic adaptation, strong familial or educational support systems and effective compensatory strategies developed during critical periods of sensory development (Hoxha et al., 2025).

However, this resilience gradually declines as age increases. As patients accumulate progressive ocular surface changes—including recurrent epithelial breakdown, pannus formation and increasing corneal irregularity—HRQoL metrics demonstrate a clear decline. Adults with more advanced stages of AAK frequently report heightened ocular discomfort, fluctuating vision, photophobia and a sense of visual unreliability, all of which can impose psychosocial burdens. The necessity for multiple medical treatments, frequent monitoring and a heightened risk of requiring repeated surgical interventions (such as glaucoma procedures or corneal surgery) adds further layers of stress, reinforcing how this disease extends far beyond static measures of acuity (Hoxha et al., 2025).

Notably, data reveal that patients who underwent early cataract extraction, often performed in attempts to optimize vision during childhood, tend to report lower satisfaction scores later in life. This paradox likely reflects the unintended consequences of disturbing the already fragile anterior segment anatomy in aniridia, with early intraocular surgery potentially accelerating the progression of LSCD and promoting glaucoma, thereby compounding visual morbidity and impacting day‐to‐day functioning (Csidey et al., 2023; Hoxha et al., 2025).

Intriguingly, detailed correlation analyses show that neither the specific *PAX6* pathogenic variant subtype nor even objective BCVA consistently predicts QoL outcomes. This underscores the multifactorial nature of well‐being in aniridia, where subjective experiences of visual fluctuation, ocular surface discomfort, glare sensitivity and the psychological impact of an unpredictable disease course often weigh more heavily than isolated genetic or visual acuity metrics (Hoxha et al., 2025).

For clinicians, these insights highlight the imperative of adopting a holistic, longitudinal approach to care. Beyond targeting anatomical preservation, there is a crucial need to address patient education, ensure robust psychosocial support and tailor management strategies not solely towards slowing disease progression but also towards optimizing functional vision and enhancing daily life satisfaction. Integrating low‐vision rehabilitation early, alongside careful counselling around the risks and timing of surgical interventions, may help mitigate the quality‐of‐life challenges inherent to living with AAK (Csidey et al., 2023; Hoxha et al., 2025) (Schoffer, unpublished).

## 7 MANAGEMENT CONSIDERATIONS

Given the intricate and multifaceted pathogenesis of AAK, effective management demands a carefully calibrated, stage‐tailored approach that accounts for the unique vulnerabilities of the aniridic ocular surface. The therapeutic priorities evolve with disease progression—ranging from preserving limbal stem cell reserves and preventing epithelial decompensation in early stages to managing established LSCD and its complications in more advanced scenarios (Latta, Figueiredo, et al., 2021; Latta, Ludwig, et al., 2021).

### 7.1 Considerations for clinical management

A key principle in the management of AAK is the strict application of a ‘no‐touch’ approach to minimize epithelial trauma and inflammatory activation. Regular use of preservative‐free artificial tears is strongly recommended to maintain ocular surface hydration and reduce mechanical stress on the fragile epithelium. All topical medications should be preservative‐free, as preservatives may exacerbate inflammation and cytotoxicity in the already compromised corneal epithelial and stromal cells. Moreover, corneal and anterior segment surgeries should be deferred whenever possible, since such interventions may accelerate AAK progression or precipitate the development of aniridia fibrosis syndrome. For patients exhibiting early signs of epithelial irregularity or recurrent micro‐erosions, autologous serum eye drops have emerged as a promising biological therapy. Rich in growth factors, vitamins and anti‐inflammatory cytokines, serum drops can promote epithelial migration and differentiation, stabilize tear film dynamics and potentially delay progression towards overt LSCD. They are particularly valuable in the interim before more invasive interventions are required, helping to maintain a smoother, less inflamed corneal surface (Fries, Náray, et al., 2024; Fries, Obst, et al., 2024).

Phototherapeutic keratectomy represents a minimally invasive option for removing superficial corneal lesions. This may also be the case in patients with congenital aniridia; nevertheless, a systematic analysis of the advantages and disadvantages of this procedure has not been analysed yet. For optimal postoperative recovery, adjunctive amniotic membrane transplantation as patch, preferably combined with autologous serum eye drops, should be taken into account (Hoxha et al., 2024).

Interestingly, a systematic review and meta‐analysis using individual patient data indicates that, in patients with AAK, Boston type 1 keratoprosthesis achieves superior anatomical success compared to graft survival after penetrating keratoplasty. In contrast, gains in best spectacle‐corrected visual acuity and rates of postoperative complications appear similar between the two procedures (Tóth, unpublished).

Clinical evidence strongly cautions against early cataract surgery in these patients unless absolutely mandated by visually significant lens opacities. While the temptation to intervene during childhood to improve amblyogenic risk profiles is understandable, intraocular surgery in the structurally fragile aniridic eye can accelerate limbal stem cell exhaustion, destabilize corneal epithelial homeostasis and precipitate secondary glaucoma. This highlights the critical importance of weighing the long‐term consequences against short‐term visual gains, with a general preference for deferring cataract extraction until clearly justified (Náray et al., 2025).

A systematic review and meta‐analysis compared the surgical outcomes of trabeculectomy and glaucoma drainage device (GDD) implantation in patients with congenital aniridia. Regarding complete success rates, trabeculectomy does not seem to provide a significant benefit over GDDs in treating aniridic glaucoma. However, the qualified success rate is greater with GDD implantation than with trabeculectomy. Both surgical approaches show similar effectiveness in lowering intraocular pressure, reducing the need for glaucoma medications and maintaining visual acuity (Tóth et al., 2025).

Ultimately, optimal care requires a multidisciplinary framework involving cornea specialists, glaucoma experts, genetic counsellors and low‐vision rehabilitation teams. Regular monitoring for subtle shifts in epithelial integrity, intraocular pressure fluctuations and patient‐reported quality‐of‐life metrics ensures that interventions are both timely and appropriately scaled, minimizing unnecessary procedures while safeguarding long‐term visual potential (Hoxha et al., 2025).

### 7.2 Management considerations in molecular research

PAX6 is a promising molecular target for future therapeutic strategies. Interventions aimed at enhancing residual PAX6 activity, modulating its downstream signalling pathways, or stabilizing the expression of key corneal differentiation markers may offer new avenues to slow or prevent the progression of AAK (Avila et al., 2025; Dorot et al., 2021; Moustardas et al., 2024; Oved et al., 2021; Suiwal et al., 2025). Furthermore, modulating FABP5 activity could help restore epithelial homeostasis, stabilize limbal stem cell function and mitigate inflammation‐driven progression of AAK (Amini, unpublished). In addition, modulation of the altered apoptotic signalling could offer a potential therapeutic approach to maintain limbal epithelial integrity and prevent further advancement of AAK (Liu, unpublished).

Looking ahead, insights gleaned from molecular investigations into the miRNA and inflammatory transcriptome of aniridia eyes open intriguing possibilities for targeted pharmacologic interventions. Restoring downregulated miRNAs like miR‐204‐5p through synthetic mimics or dampening overexpressed miRNAs such as miR‐138‐5p with specific inhibitors could theoretically rebalance angiogenic, apoptotic and proliferative signalling cascades (Abbasi et al., 2024; Amini et al., 2025) (Suiwal, unpublished). Likewise, biologics designed to inhibit key inflammatory pathways—such as IL‐17 or TNF antagonists, already established in systemic autoimmune diseases—might one day be adapted for topical or periocular use to quell the chronic subclinical inflammation that perpetuates LSCD in aniridia (Stachon, unpublished) (Table 1).

**TABLE 1:** Clinical and in vivo confocal microscopic (IVCM) findings and molecular mechanisms driving aniridia‐associated keratopathy (AAK) progression and potential therapeutic targets in *PAX6*‐related congenital aniridia.

Clinical findings	IVCM findings	Molecular mechanisms	Potential therapeutic targets
Progressive limbal epithelial stem cell deficiency with corneal neovascularization, conjunctivalization, epithelial defects, ulcer, pannus formation (Lagali et al., 2018)	Disruption of orderly corneal epithelial turnover and stratification (Lagali et al., 2018)	PAX6 haploinsufficiency (Latta et al., 2019) Reduced KRT3, KRT12 differentiation markers in limbal epithelial cells (LECs) (Latta et al., 2018) Disruption of retinoic acid signalling (dimished ADH7 and ALDH1A1) (Latta et al., 2019) miR‐204‐5p downregulation (Latta, Figueiredo, et al., 2021; Latta, Ludwig, et al., 2021) miR‐138‐5p upregulation (Nastaranpour et al., 2025)	Upregulation of residual PAX6 activity Modulation of PAX6 downstream effectors Stabilization of critical corneal differentiation markers miR‐204‐5p upregulation/restoring/mimics miR‐138‐5p downregulation/antagonizing/inhibitors
Ocular surface inflammation (Lagali et al., 2018)	Increased density and maturity of Langerhans cells (Csorba et al., 2024)	IL‐17 and TNF upregulation (Stachon, unpublished)	(1) IL‐17 and TNF inhibition/downregulation
Corneal sensitivity changes	CNFD, CNFL, CTBD decrease CNFW increase (Csorba et al., 2024)	Unknown	Unknown
Corneal nerve alterations		Unkonwn	Unknown
Stromal haze and scarring	Decreased keratocyte density Hyperreflective punctate deposits Increased density and maturity of Langerhans cells (Csorba et al., 2024)	Alterations of PAX6, COL1A1, COL5A1, ALDH3A1, KER and α‐SMA levels (Li et al., 2025a, 2025b, 2025c, 2025d) LPS markedly elevates expression of pro‐inflammatory cytokines, particularly IL‐1β and IL‐6 (Trusen et al., 2024) Oxidative stress results in heightened activation of Nrf2, SOD1 and PRDX6 (Zimmermann et al., 2025)	Upregulation of residual PAX6 activity Inflammatory pathway modulation Antioxidative pathway modiulation
Endothelial changes	Lower cell diameter Lower Clark‐Evans‐Index Endothelial deposits (Csidey et al., 2025).	Unknown	Unknown

Abbreviations: ADH7, alcohol dehydrogenase 7; ALDH1A, Aldehyde Dehydrogenase 1 Family Member A; ALDH3A1, Aldehyde Dehydrogenase 3 Family Member A1; CNFD, corneal nerve fibre density; CNFL, corneal nerve fibre length; CNFW, corneal nerve fibre width; COL1A1, collagen type I alpha 1 chain; COL5A1, collagen type V alpha 1 chain; CTBD, corneal nerve branch density; IL‐17, interleukin 17; IL‐1β, interleukin‐1 beta; IL‐6, interleukin‐6; KER, keratocan; KRT12, keratin 12; KRT3, keratin 3; miR, microRNAs; Nrf2, nuclear factor erythroid 2–related factor 2; PAX6, paired box gene 6; PRDX6, peroxiredoxin 6; SOD1, superoxide dismutase 1; TNF, tumour necrosis factor; α‐SMA, alpha‐smooth muscle actin.

In addition to these pharmacologic advances, the field of ocular surface regenerative medicine offers hope for more definitive solutions. Techniques such as ex vivo expansion of autologous or allogeneic LECs, cultivated oral mucosal epithelial transplantation, and the engineering of bioactive scaffolds seeded with stem or progenitor cells are under active exploration. These strategies aim not merely to palliate symptoms but to rebuild a sustainable limbal niche capable of restoring corneal clarity and function. In the future, combining such cellular therapies with molecular modulators tailored to the individual patient's genetic and transcriptomic profile could usher in a truly personalized approach to managing AAK (Latta, Figueiredo, et al., 2021; Latta, Ludwig, et al., 2021; Schlötzer‐Schrehardt et al., 2021).

## 8 CONCLUSIONS

In summary, AAK stands as a compelling example of a multifactorial ocular surface disorder situated at the crossroads of genetic developmental defects, chronic low‐grade inflammation and intricate disruptions in cellular cross‐communication. Unlike many isolated corneal diseases, AAK's origins are deeply rooted in the foundational architecture of the eye, with *PAX6* haploinsufficiency setting into motion a cascade of anomalies that ripple across nearly every anterior segment structure.

High‐resolution imaging modalities, particularly IVCM, have provided unprecedented windows into the microstructural perturbations of the cornea in aniridia—revealing profound alterations in subbasal nerve plexus density, keratocyte organization, Langerhans cell infiltration and endothelial morphology (Csidey et al., 2025; Csorba et al., 2024; Lagali et al., 2020). These features not only chart the extent of anatomical compromise but also correlate with the evolving clinical findings of LSCD and progressive vision loss.

In parallel, sophisticated molecular investigations, ranging from miRNA profiling to whole‐transcriptome sequencing, continue to unravel the complex regulatory networks that sustain and exacerbate disease progression. The discovery of specific miRNA dysregulations—such as the downregulation of miR‐204‐5p and upregulation of miR‐138‐5p—and the activation of inflammatory cascades including IL‐17 and TNF signalling underscore that AAK is not merely a static consequence of faulty development but a dynamic, actively modulated process. This evolving understanding highlights tangible molecular targets that may be exploited for future disease‐modifying interventions (Abbasi et al., 2024; Amini et al., 2025; Hsu et al., 2025) (Suiwal, Stachon, unpublished).

Beyond the bench, these insights carry profound implications for patient care. They argue for a more nuanced, multidisciplinary approach, combining vigilant ophthalmic monitoring with genetic counselling, tailored medical therapy and psychosocial support. They also reinforce the urgency of refining surgical decision‐making, particularly around the timing of cataract extraction and glaucoma interventions, to mitigate unintended acceleration of corneal surface deterioration (Náray et al., 2025; Tóth et al., 2025).

Looking ahead, the promise of precision medicine in AAK is increasingly within reach. Advances in topical or locally delivered molecular modulators, coupled with breakthroughs in regenerative strategies—such as ex vivo expanded limbal stem cell grafts or bioengineered niche scaffolds—offer the possibility not only of slowing disease progression but perhaps even of restoring sustainable corneal clarity and function.

Ultimately, furthering our grasp of these deeply interwoven mechanisms is crucial, not only to preserve anatomical transparency but also to profoundly enhance the lived experiences of patients with congenital aniridia. Through this lens, each new molecular pathway elucidated and each microstructural nuance decoded brings us a step closer to interventions that can transform QoL, offering hope where therapeutic options have long been limited.
